# Use of tissue adhesives in ophthalmology

**DOI:** 10.4103/0301-4738.55059

**Published:** 2009

**Authors:** Rajesh Sinha, Chandrashekhar Kumar, Namrata Sharma

**Affiliations:** S-7, Dr. R. P. Centre for Ophthalmic Sciences, AIIMS, New Delhi

Tissue adhesives can be divided into synthetic adhesives (*e.g.,* cyanoacrylate derivatives) and biologic adhesives (*e.g.,* fibrin-based adhesives). Cyanoacrylate derivatives are compounds with very high tensile strength that rapidly polymerize on contact with basic substances such as water or blood to form a strong bond. Because they are synthetic and nonbiodegradable, they are usually used on an external surface and may induce an inflammatory foreign body reaction, including neovascularization and tissue necrosis. Fibrin-based adhesives, by contrast, have a lower tensile strength and slower polymerization, but, being biologic and biodegradable, they may be used under a superficial covering layer (*e.g.,* conjunctiva, amniotic membrane) and induce minimal inflammation. These adhesives have been used in ophthalmology to treat various ocular conditions. In the present article, we have made an attempt to include most of the studies performed in ophthalmology with the tissue adhesives.

## Retina

### Retinal detachment

**Hartnett *et al***. (*Retina 1998;18(2):125-9*) reported the experience with cyanoacrylate glue in posterior retinal breaks associated with retinal detachments in four consecutive pediatric patients who underwent vitreoretinal surgery for retinal detachment. In three of the four patients, successful retinal reattachment with visual function was achieved by vitreoretinal surgery and cyanoacrylate glue placed on the apposed edges of posterior retinal breaks or used to plug a break (postoperative follow-up was 1.5-2 years). In two successful cases, the glue was applied onto the break while the retina was detached, which resulted in closure of the retinal breaks and reattachment of the retina.

**Sheta *et al***. (*Am J Ophthalmol. 1986;102(6):717–22*) evaluated the use of trans-vitreal cyanoacrylate retinopexy during vitreous surgery in the treatment of experimental rhegmatogenous retinal detachment in rabbit eyes. The chorioretinal adhesions produced with cyanoacrylate tissue adhesive were compared with those produced by trans-scleral retinal cryopexy and were found to be more rapid in onset as well as stronger. An exaggerated tissue response adjacent to the cyanoacrylate site suggested a potential toxic chemical or thermal reaction, or both, to the tissue adhesive.

**Ricci *et al***. (*Acta Ophthalmol Scand. 2001;79(5):506-8*) performed an experimental sutureless scleral buckling using a tissue adhesive glue to fixate a silicone band to the sclera. An encircling band of silicone, which is generally anchored to the sclera itself, was sutured in 36 rabbit eyes to three small silastic patches that had been glued to the sclera, at the level of the equator, using octyl 2-cyanoacrylate tissue adhesive. The band was tightened to produce buckling of the sclera and its ends were glued together using the same adhesive. Examination of the eyes from 15 days to 6 months after surgery revealed that the buckle was stable, with no signs of slippage, in 33 eyes. In the remaining 3 (one examined after 15 days, 2 examined after 45 days), one of the three support patches had detached but there was still no evidence of slippage.

### Macular hole

**Tilanus *et al***. (*Int Ophthalmol. 1994-1995;18(6):355-8*) used, in addition to vitrectomy and gas tamponade, a tissue glue to stimulate adhesion of the elevated cuff of neurosensory retina surrounding a full-thickness macular hole in 15 eyes of 13 patients, with stage 3 and 4 macular holes. All of the 13 uncomplicated cases showed complete closure of the macular hole. In one case the visual acuity decreased one line in spite of a funduscopically closed hole, and in two cases visual acuity remained the same despite closure. Increased visual acuity was seen in ten cases (76%), eight of which improved more than two lines.

### Conjunctival closure following pars plana vitrectomy

**Mentens *et al***. (*Am J Ophthalmol. 2007;144(1):128-31*) successfully used fibrin glue in conjunctival closure following 20 gauge pars plana vitrectomy and reported a shorter duration of conjunctival congestion (p=0.0471), and discomfort of the eye (p=0.0376).

### Transforming growth factor beta (TGF-beta) as tissue glue

**Smiddy *et al***. (*Arch Ophthalmol. 1989;107(4):577-80*) studied transforming growth factor beta (TGF-beta) for a possible role as a bioactive substance for inducing localized chorioretinal wound healing along the edge of a retinal tear. The edges of the retinal tear treated with TGF-beta were adherent to the underlying Bruch′s membrane via localized fibrous tissue without apparent effects elsewhere suggesting that TGF-beta may have a potential role as an alternative means for inducing a chorioretinal adhesion in the treatment of retinal tears.

## Cornea

### Amniotic Membrane Patches

**Kitagawa *et al***. (*Am J Ophthalmol. 2009 May 22*) reported use of hyperdry amniotic membrane (AM) patches using a tissue adhesive for corneal perforations and bleb leaks. Five eyes of 5 patients (glaucoma bleb leaks, 2 eyes; corneal perforations, 3 eyes) were treated with a single-layer patch of dried AM using a biological tissue adhesive. The tissue adhesive was applied to the epithelial side of the dried membrane cut into desired size and shape and then was positioned to cover the conjunctival bleb leak site or corneal perforation lesion using forceps. A therapeutic hydrogel contact lens was placed as a bandage. Bleb leaks or corneal perforations were repaired successfully within 21 days in all 5 cases. There were no remarkable adverse effects, and there was no recurrence of bleb leak or corneal perforation.

### Fibrin glue (FG)-assisted augmented amniotic membrane transplantation

**Kim *et al***. (*Cornea 2009;28(2):170-6*) evaluated and reported the efficacy of fibrin glue (FG)-assisted augmented amniotic membrane transplantation (AMT) in 10 patients with large noninfectious corneal perforations more than 2 mm in diameter. A 5- or 7 layered “augmented amniotic membrane” was constructed by applying FG to each sheet of AM to repair the corneal perforation. The augmented AM was designed 0.5 mm larger than the diameter of the perforation and was transplanted onto the perforation site with 10-0 nylon suture. If needed, additional overlay AM was sutured on top. The integrity of the eyeball was maintained in all the patients after the procedure and had well-formed deep anterior chambers and 90% of patients showed complete epithelialization over the AM. The mean reepithelialization time was 14.9±4.9 days (range, 10-24 days). No eye showed evidence of infection or recurrent corneal melting during the follow-up period.

### Corneal Perforations

**Weiss *et al***. (*Ophthalmology1983;90(6):610-5*) reported that eighty patients with either corneal perforation or impending perforation were treated with the application of tissue adhesive and it remained in place on the average of 50 days. Forty-four percent of these cases healed with the application of glue alone. Complications occurred in nine patients (11%). Two developed marked increase in intraocular pressure which was controlled with antiglaucoma medications and seven developed corneal infiltrates. Five of the infiltrates were culture-proven bacterial infections occurring on an average of 73 days after gluing.

**Sharma *et al***. (*Ophthalmology 2003;110(2):291-8*) compared the efficacy of fibrin glue (Group 1; n=19) and N-butyl-2-cyanoacrylate glue (Group 2; n=22) in corneal perforations. Fifteen (79%) eyes had successful healing of corneal perforation in group 1, compared with 19 (86%) eyes in group 2 (*P* > 0.05) at 3 months’ follow-up. Corneal perforation healed within 6 weeks in 12 (63%) eyes in group 1 and 7 (31.8%) eyes in group 2 (*P* < 0.05). Reapplication of glue was required in six (31.5%) eyes in group 1 and seven (31.4%) eyes in group 2 during the first 3 months of follow-up. The mean number of applications per eye was 1.37 in group 1 and 1.36 in group 2. An increase in deep corneal vascularization was observed in 2 (10.5%) eyes in group 1 and 10 (45.5%) eyes in group 2 (*P* < 0.05). Giant papillary conjunctivitis occurred in one (5%) eye in group 1 and eight (36.4%) eyes in group 2 (*P* < 0.05). They concluded that both tissue adhesives are effective in the closure of corneal perforations up to 3 mm in diameter. Fibrin glue provides faster healing and induces significantly less corneal vascularization, but it requires a significantly longer time for adhesive plug formation.

**Siatiri *et al***. (*Cornea2008;27(9):988-91*) used FG and soft contact lens to seal corneal perforations of 0.6-3 mm (mean = 1.88 mm). The etiologies of corneal perforation were post-infectious keratitis in 11 and noninfectious in 7. Fifteen (83.3%) eyes had successful healing of corneal perforation after 3 months. All the patients who failed had corneal perforation larger than 2 mm in diameter. The success rate was higher in corneal perforation of ≤ 2 mm in diameter. No case developed giant papillary conjunctivitis or secondary glaucoma. It provided fast healing with low rate of corneal vascularization.

### Pterygium

**Uy *et al***. (*Ophthalmology2005;112(4):667-71*) compared the efficacy and safety of fibrin glue and suturing for attaching conjunctival autografts among 22 patients undergoing pterygium excision. A superior conjunctival autograft was harvested and transferred onto bare sclera after pterygium excision. Fibrin glue was used to attach the autograft in 11 eyes and 10-0 nylon suture was used to attach the autograft in 11 eyes. The patients were followed up for 2 months. All conjunctival autografts in both groups were successfully attached and were intact after 2 months. The average operating time for the fibrin glue group was significantly shorter (P<0.001). Postoperative symptoms were fewer for the fibrin glue group than the suture group.

**Marticorena *et al***. (*Cornea2006;25(1):34-6*) evaluated the efficiency and safety of using a fibrin adhesive to avoid the need for sutures during conjunctival autograft surgery for primary pterygium in 20 eyes. The time of surgery was 15 to 20 minutes. During the postoperative course, none of the patients felt pain, and only 25% had mild sensation of the presence of a foreign body. In 90% of the patients, the conjunctival autograft was fixed (mean follow-up: 26.05±3.15 weeks) with no case of recurrence of pterygium.

**Bahar *et al***. (*Cornea2006;25(10):1168-72*) compared the short-term results of conjunctival closure in pterygium surgery using fibrin adhesive versus Vicryl (polyglactin) sutures in 65 eyes. Surgery consisted of the bare sclera technique combined with intraoperative mitomycin C. The average operative time was 16 minutes (range, 14-16 minutes) in the fibrin glue group and 20 minutes (range, 20–29 minutes) in the Vicryl suture group (*P* < 0.05). Significantly less pain, photophobia, foreign body sensation, irritation, epiphora, itching, local hyperemia, conjunctival chemosis, and dry eye were noted in the subjects treated with glue (P < 0.05) and no complication was noted during the 3-week follow-up.

**Bahar *et al***. (*Curr Eye Res. 2007;32(5):399-405*) compared the long-term results of conjunctival closure with fibrin adhesive (n = 42) or Vicryl sutures (n = 39) in 81 eyes with primary nasal pterygium and followed up for 1 year. Surgery in all patients consisted of the bare sclera technique combined with intraoperative administration of mitomycin C 0.02%. There were no complications during follow-up period in the glue-treated patients; one patient in the suture group had a medically treatable corneal dellen. At the end of follow-up, recurrent pterygium developed in 11.9% eyes of the fibrin-glue group and in 7.7% eyes of the Vicryl-suture group (p < 0.05).

**Jain *et al***. (*Cornea2008;27(1):94-9*) reported the use of fibrin glue for attaching the human amniotic membrane graft with a tuck-in technique after primary pterygium excision in 12 eyes. The bare sclera was covered with an oversized human amniotic membrane graft by using fibrin glue for graft adherence. The edges of the graft were tucked underneath the adjacent free margin of conjunctiva on 3 sides. The amniotic membrane graft adhered successfully in 11 patients. Average surgical time was 15.5 minutes (range, 13-21 minutes). The postoperative period was generally comfortable. Epithelialization of the graft was rapid, occurring within 7 days in 11 eyes. In 1 patient, the graft was found dislodged partially on the first postoperative day. Eleven eyes showed no recurrence at the end of 1-year follow-up.

**Karalezli *et al***. (*Br J Ophthalmol.2008;92(9):1206-10*) compared the use of fibrin glue versus sutures for fixating conjunctival autografts in 50 patients undergoing pterygium excision. In the fibrin glue group (n=25), the mean operation time was 15.7 ± 2.4 min (range 12-18 min) and in the suture group (n=25) it was 32.5 ± 6.7 min (range 25-40 min) (p<0.001). The intensity of the postoperative pain, foreign-body sensation, irritation and epiphora were significantly lower in the fibrin glue group than in the suture group (p<0.001). The intensity of itchy sensation at the first two postoperative visits was lower among patients in the fibrin glue group (five patients, 20%) than in the suture group (12 patients, 48%) (p<0.05). Two patients in the fibrin glue group had partial graft dehiscence; these grafts were successfully reattached with fibrin glue. At the end of follow-up, pterygium recurrence was observed in one eye (4%) in the fibrin glue group and in three eyes (12%) in the suture group (p<0.05).

**Benyamini *et al***. (*Cornea2008;27(8):911-5*) compared two surgical approaches to conjunctival flap placement during pterygium surgery using a biologic adhesive: single rotational flap (group A, 19 eyes) and double sliding flaps (group B, 15 eyes). The patients were followed for 24 weeks. In group A, 1 (5.3%) flap was lost because of ischemia, whereas all the others remained in position and became fully integrated. In group B, 4 (26%) flaps did not retain their primary position.

**Srinivasan *et al***. (*Br J Ophthalmol.2009;93(2):215-8*) compared the degree of conjunctival autograft inflammation, subconjunctival haemorrhage (SCH) and graft stability following the use of sutures or FG during pterygium surgery in 40 eyes. The degree of inflammation was significantly less with FG than with sutures at 1 month (p = 0.019) and 3 months (p = 0.001) postoperatively. No significant difference was found for inflammation at 1 week postoperatively. Conjunctival grafts secured with FG were as stable as those secured with sutures. No significant difference was found in degree of postoperative SCH between the groups.

### Wound closure after cataract surgery

**Mester *et al***. (*J Cataract Refract Surg.1993;19(5):616-9*) described astigmatism after phacoemulsification with posterior chamber lens implantation in small incision technique with fibrin adhesive for wound closure. They conducted a comparative study of 385 consecutive patients; 167 received only fibrin glue for wound closure and 218 had the single-stitch procedure. No complication was observed in either group. Surgically induced astigmatism was smaller in the fibrin group.

**Grewing *et al***. (*Ophthalmic Surg. 1994;25(7):446-8*) evaluated the efficacy of using a modified wound-closure technique in cataract surgery to reduce preexisting against-the-rule (ATR) astigmatism. Seventy-seven eyes received a radial 10-0 nylon suture in the axis of the preexisting ATR cylinder, combined with an application of fibrin glue to stabilize the wound. A control group of 76 patients with comparable preoperative ATR astigmatism was operated on in the same manner, but only fibrin glue and no suture was used for wound closure. The mean induced astigmatism in these two groups differed by 0.42 diopters (P < 0.05). In the cases with preoperative astigmatism greater than 1.00 D, the difference between the two groups was 0.73 (P < 0.05).

### Glued IOL

**Agarwal *et al***. (*J Cataract Refract Surg.2008;34(9):1433-8*) reported a new surgical technique that uses biological glue to implant a posterior chamber intraocular lens (PC IOL) in eyes with a deficient or absent posterior capsule. Two partial-thickness limbal-based scleral flaps were made 180 degrees apart diagonally, and the haptics of the PC IOL were externalized to place them beneath the flaps. Fibrin glue was used to attach the haptics to the scleral bed, beneath the flap. This simple method of PC IOL implantation requires no specially designed haptics. It provides good flap closure and IOL centration.

**Nair *et al***. (*Eye Contact Lens2009;35(4):215-7*) reported a patient of retinitis pigmentosa with spontaneous bilateral anterior in-the-bag subluxation of PCIOL who was managed by IOL explantation followed by fibrin-glue-assisted sutureless PCIOL implantation. Two partial thickness limbal-based scleral flaps were created about 1.5 mm from the limbus under which sclerotomies were made. Intraocular lens explantation along with capsular bag was performed through the corneo-scleral tunnel incision. Single-piece rigid polymethylmethacrylate 6.5-mm optic IOL was introduced through the limbal wound with a McPherson forceps, both the IOL haptics were externalized under the scleral flap. The haptic ends were tucked in the scleral tunnel made with the 26G needle. Scleral flaps and the conjunctiva were closed with the fibrin glue. Preoperative best corrected visual acuity was 20/80 in the right and 20/120 in the left eye. Patient gained a best corrected visual acuity of 20/30 in both the eyes, with a bilateral stable PCIOL and clear cornea.

### For epithelial ingrowth after laser *in situ* keratomileusis (LASIK) enhancement

**Narvaez *et al***. (*Cornea2006;25(9):1115-7*) reported a case of clinically significant post- LASIK epithelial ingrowth successfully treated with a combined technique of mechanical debridement, flap suturing, and fibrin glue application. No recurrence was found during a 15-month follow-up period. No adverse effects were seen with this approach.

### Harvesting keratolimbal allografts from corneoscleral buttons

**Lim *et al***. (*Br J Ophthalmol.2008;92(11):1550-1*) used cyanoacrylate glue to fix the two halves of donor rim on a sterile rubber block (the under surface of the donor punch) after cutting the donor tissue for penetrating keratoplasty for limbal stem cell dissection. This composite provided stability to the donor rim allowing lamellar dissection of the limbal tissue without damaging the limbal epithelium.

### Band shaped keratopathy (BSK)

**Esquenazi *et al***. (*Ophthalmic Surg Lasers Imaging2008;39(5):418-21*) reported a case of recurrent BSK in which they removed the calcified lesion surgically and then performed an 8-mm, 100-micron trephination and created a 360 degrees lamellar peripheral corneal dissection pocket. After covering the denuded corneal surface, the edges of the amniotic membrane were introduced into the pocket and secured using fibrin sealant. Additional amniotic membrane was glued to the nasal and temporal corneal areas and a collagen shield was applied. There was complete wound healing in 10 days and a stable ocular surface was restored without pain or inflammation.

### Antibacterial analysis of Ethyl-cyanoacrylate

**de Almeida Manzano *et al***. (*Cornea2006;25(3):350-1*) analyzed the antimicrobial properties of ethyl-cyanoacrylate *in vitro* against *Staphylococcus aureus, coagulase-negative Staphylococcus, Streptococcus pyogenes, Streptococcus pneumoniae, Pseudomonas aeruginosa, Escherichia coli* and *Enterococcus faecalis*. One drop of the glue was dropped directly into the nutrient broth and the plates were incubated for 24 hours. Bactericidal activity of the glue was verified by sampling inhibition zones when present. The samples were plated in blood agar and analyzed after 24 and 48 hours. The ethyl-cyanoacrylate inhibited the growth of every gram-positive microorganism tested and showed bactericidal effect over 70% for all of them. Among the gram-negative microorganisms, only the *E. coli* and the *E. faecalis* had its growth inhibited.

## Keratoplasty

### Lamellar keratoplasty

**Duarte *et al***. (*Cornea 2007;26(9):1127-8*) reported a case where fibrin glue was applied with a modified approach to secure the graft to the host bed in lamellar keratoplasty. The glue components were applied separately: the fibrin component on the stromal bed and thrombin component on the stromal side of the lamellar graft. Postoperatively, best-corrected visual acuity 4 months after surgery was 20/30. The graft remained clear and well positioned at 9 months follow up after surgery. They concluded that components of the glue can be applied separately in a lamellar keratoplasty, allowing more time to position the graft on the stromal bed.

**Kaufman *et al***. (*Ophthalmology2003;110(11):2168-72*) determined whether a fibrin adhesive can facilitate the performance of sutureless lamellar keratoplasty and attachment of AM to bare sclera. Six patients were studied, 5 of whom underwent lamellar keratoplasty and 1 received an amniotic patch. In 5 patients, the epithelium was removed from the corneal surface, a free cap, 200μ thick, was cut with a microkeratome, and FG was applied to the stromal bed. A 200μ thick, microkeratome-cut lamellar graft was placed in the stromal bed without sutures, and a bandage soft contact lens was applied. The lens was left in place for 1 week and then removed. In 1 patient, the adhesive was applied to bare sclera for attachment of AM after removal of a conjunctival melanosis. All patients were followed up for 3 months after surgery. All 5 lamellar grafts healed and remained clear; the AM graft also adhered well to the bare sclera.

### Deep Anterior Lamellar Keratoplasty

**Narendran *et al***. (*Cont Lens Anterior Eye 2007;30(3):207-9*) described a case where Deep Anterior Lamellar Keratoplasty (DALK) was performed using overlay sutures and fibrin glue alone, without direct suture to the corneal button. All securing sutures were removed 4 weeks after the surgery. Six months post surgery, the graft was clear and the patient rehabilitated by wearing acceptable astigmatic spectacle correction.

### Squint surgery

**Biedner *et al***. (*Ophthalmic Surg Lasers 1996;27(11):967*) described six patients undergoing bilateral symmetric strabismus surgery; for each subject, incisions were closed with Vicryl in one eye and glue in the other. The conjunctival closure with the Vicryl suture resulted in increased discomfort and inflammation during the early postoperative period compared with fibrin glue; this difference disappeared after 14 days.

## Glaucoma

### Bleb leakage

**Asrani *et al***. (*Ophthalmology1996;103(2):294-8*) evaluated autologous fibrin tissue glue (AFTG) in the treatment of bleb leaks. Successful healing of the leaks was obtained in 9 of the 12 episodes in which AFTG was used. However, there were no statistically significant differences between AFTG and the other treatment modalities.

**Grewing *et al*** (*Ophthalmic Surg Lasers1997;28(2):124-7*) described two patients with hypotony that occurred after trabeculectomy in one case and after combined cataract and glaucoma surgery in another case. Temporary tamponade of the scleral flap was achieved by subconjunctival injection of fibrin sealant. After the fibrin sealant was applied, the choroidal detachment resolved and intraocular pressure increased to normal.

### Fibrin glue-assisted glaucoma drainage device surgery

**Kahook *et al***. (*Br J Ophthalmol. 2006;90(12):1486-9*) described the use of FG as a suture substitute for portions of glaucoma drainage device (GDD) surgery and compared with traditional suture material. No significant difference was observed in IOP levels at any time point between the two groups. Conjunctival inflammation was more pronounced in the suture group (p=0.002). The mean time of surgery was significantly less for the glue-assisted group (p<0.001).

**Valimaki** (*Acta Ophthalmol Scand. 2006;84(3):372-4*) reported the use of fibrin glue to prevent a leak of aqueous around the tube in the immediate postoperative period after glaucoma drainage implant (GDI) surgery in 42 eyes. Fibrin glue was used over the scleral flap intraoperatively in every eye with peritubular leakage. All 11 eyes maintained an intraocular pressure (IOP) of ≥16 mmHg in the immediate postoperative phase. No complication or Seidel-positive aqueous leak was observed during the follow-up period.

## Occuloplasty

### Glue blepharorrhaphy

**Sonmez *et al***. (*Eur J Ophthalmol. 2008;18(4):529-31*) reported the effectiveness of cyanoacrylate glue blepharorrhaphy in immobilized patients with recalcitrant exposure keratopathy. Temporal two thirds of upper eyelid eyelashes were glued to lower eyelid skin with tissue adhesive n-butyl-2-cyanoacrylate after application of a contact lens in 12 eyes of 9 patients. All the corneal ulcers healed within 4 to 11 days (mean: 5.5 days). Blepharorrhaphy opened spontaneously in 4 to 21 days (mean: 8.6 days).

### Sealing CSF leakage after Orbital exenteration

**Yuen *et al***. (*Ophthal Plast Reconstr Surg.2008;24(3):238-40*) reported successful sealing of leakage of CSF complicated by orbital exenteration by the application of cyanoacrylate tissue glue.

### Eyelid skin grafts

**Shorr *et al***. (*Ophthal Plast Reconstr Surg.1991;7(3):190-3*) reported a clinical series of 18 patients in which eyelid skin grafts were placed with a combination of sutures and cyanoacrylate tissue glue. No complication was encountered. The postoperative course and results were identical to the skin grafts closed with suture alone.

### Mucous membrane graft

**Watts *et al***. (*Ophthalmic Surg. 1992;23(10):689-90*) reported using FG in place of sutures to place a full-thickness mucosal graft into a defect created in a scarred fornix of an ophthalmic socket. They concluded that this technique minimizes tissue trauma and aids hemostasis and healing.

**Gallemore *et al***. (*Ophthal Plast Reconstr Surg.1999;15(3):210-2*) described a technique to secure a mucous membrane graft to a custom conformer during reconstruction of the conjunctival fornices and socket. Cyanoacrylate-based tissue glue was used instead of sutures to secure the mucous membrane graft to the conformer. The adhesion between the graft and the conformer weakened over time, permitting easy removal of the conformer from the socket 6 to 12 weeks postoperatively. No complication was encountered in any of the six patients in whom this technique was used.

